# Effect of thermocycling, mechanical loading on longevity of endodontically treated teeth restored with zirconia, fiber posts

**DOI:** 10.6026/973206300212012

**Published:** 2025-07-31

**Authors:** Shrutika Jadhav, Jasmine Marwaha, Divya Batra, Pooja Arora, Ashtha Arya, Nirman Das

**Affiliations:** 1 Department of Conservative Dentistry and Endodontics, D.Y. Patil Dental School, Charholi Budruk, Lohegaon, Pune, India; 2 Department of Conservative Dentistry and Endodontics, National Dental College and Hospital, Derabassi-140507, Punjab, India; 3 Department of Prosthodontics, Taif University, Kingdom of Saudi Arabia; 4 Department of Conservative Dentistry and Endodontics, SGT Dental College, Hospital and Research Institute, SGT University, Gurugram, Haryana- 122505, India; 5 Department of Periodontology and Oral Implantology, Kalinga Institute of Dental Sciences, Bhubaneswar, Odisha, India

**Keywords:** zirconia posts, fiber posts, endodontically treated teeth, *in vitro* study, bond strength, fatigue resistance, thermocycling, mechanical loading, post retention, restoration longevity, dental materials, adhesive failure, post-endodontic restoration

## Abstract

The comparative performance of zirconia and fiber posts in endodontically treated teeth under simulated oral conditions, show significant differences in fatigue resistance and bond strength degradation. Zirconia posts initially exhibited superior bond strength; fiber posts maintained better long-term stability under thermocycling and mechanical loading conditions, with implications for clinical longevity and restoration success rates.

## Background:

Endodontically treats teeth (ETT) present unique challenges in restorative dentistry due to structural modifications that occur during root canal treatment and the subsequent need for intra-radicular reinforcement [1]. The restoration of these teeth typically requires post and core systems to provide adequate retention and support for the final restoration, particularly in cases with substantial coronal tooth structure loss [2]. The selection of appropriate post materials and restoration protocols has become increasingly critical as clinicians seek to maximize the longevity of endodontic restorations while minimizing the risk of catastrophic failure. Two primary categories of post materials dominate contemporary endodontic practice: fiber-reinforced composite posts and ceramic posts, particularly those fabricated from zirconia. Fiber posts, typically composed of glass or carbon fibers embedded in a resin matrix, have gained popularity due to their elastic modulus approximating that of dentin and their reported ability to create more favorable stress distribution patterns [3, 4-5]. Conversely, zirconia posts offer superior mechanical properties, including high flexural strength and fracture toughness, making them attractive for high-stress clinical situations. The clinical performance of post and core restorations is significantly influenced by the complex oral environment, which subjects these restorations to repetitive mechanical stresses and thermal fluctuations [4, 6]. Thermocycling, which simulates the temperature variations experienced during normal oral function, has been extensively used in laboratory studies to evaluate the durability of dental materials and bonding interfaces [7, 8]. Studies have demonstrated that thermocycling can significantly affect the bond strength between various dental materials, with reported reductions ranging from 15% to 40% depending on the material combination and testing parameters. Mechanical fatigue testing has emerged as a crucial methodology for evaluating the long-term performance of dental restorations under clinically relevant loading conditions. Unlike static load testing, which provides information about ultimate failure loads, fatigue testing simulates the repetitive nature of masticatory forces and can reveal failure mechanisms that may not be apparent under single-load conditions [9, 10, 11, 12-13]. Research has shown that the fatigue behavior of post and core systems can vary significantly based on material properties, geometric factors and interfacial characteristics [10, 12]. Recent investigations have highlighted the importance of considering both thermal and mechanical aging when evaluating the performance of endodontic restorations. The synergistic effects of these aging processes may accelerate degradation mechanisms that would not be apparent when these factors are considered independently [14]. Furthermore, the mode of failure in post and core systems has significant clinical implications, as restorable failures above the cementoenamel junction are preferable to catastrophic root fractures that necessitate tooth extraction [15 and 16]. Therefore, it is of interest to comparative effect of thermocycling, mechanical loading on longevity of endodontically treated teeth restored with zirconia, fiber posts.

## Materials and Methods:

## Specimen preparation:

Sixty freshly extracted human maxillary canines with similar dimensions and intact roots were selected for this study following institutional review board approval. Teeth were stored in 0.5% chloramine-T solution at 4°C for no more than three months prior to use. Root canal treatment was performed using a standardized protocol with nickel-titanium rotary instruments (ProTaper Universal, Dentsply Sirona) and canals were obturated with gutta-percha and AH Plus sealer (Dentsply DeTrey). After one week of storage in 100% humidity at 37°C, crowns were sectioned 2 mm coronal to the cementoenamel junction to simulate clinical scenarios with extensive coronal destruction. Post space preparation was accomplished using the manufacturer's recommended drills to a depth of 10 mm, leaving 4 mm of apical filling material. The prepared specimens were randomly divided into two groups (n=30 each): Group FP (fiber posts) received glass fiber posts (D.T. Light-Post, VDW) and Group ZP (zirconia posts) received custom-milled zirconia posts (Cercon ht, Dentsply Sirona). Posts were cemented using dual-cure resin cement (RelyX Ultimate, 3M ESPE) following manufacturer's instructions, with excess cement carefully removed and light-cured for 40 seconds from multiple directions.

## Core build-up and crown fabrication:

Composite resin cores (Filtek Z350 XT, 3M ESPE) were built to a standardized height of 5 mm with a 6-degree total occlusal convergence and 1 mm chamfer finish line. All-ceramic crowns were fabricated using a CAD/CAM system (CEREC, Dentsply Sirona) from lithium disilicate ceramic blocks (IPS e.max CAD, Ivoclar Vivadent) with uniform wall thickness of 1.5 mm [13, 14, 15, 16- 17]. Crowns were cemented using the same resin cement system with appropriate surface treatments according to manufacturer protocols.

## Aging protocols:

Each group was further subdivided into three subgroups (n=10 each): control (no aging), thermocycling only (TC) and combined thermocycling and mechanical loading (TC+ML). Thermocycling was performed using a thermal cycling machine (Thermocycler 1200, Vötsch Industrietechnik) for 5,000 cycles between 5°C and 55°C with a dwell time of 30 seconds and transfer time of 5 second. This protocol simulates approximately six months of clinical service based on established correlations. Mechanical loading was applied using a computer-controlled chewing simulator (CS-4.8, SD Mechatronik) with specimens mounted at 30 degrees to the loading axis . A cyclic load of 100 N was applied at 2 Hz frequency for 1.2 million cycles, simulating approximately two years of clinical function. Loading was applied to the lingual aspect of the crown through a 6 mm diameter flat antagonist to simulate contact with opposing dentition.

## Bond strength evaluation:

Following aging protocols, specimens designated for bond strength evaluation underwent push-out testing using a universal testing machine (Instron 5566, Instron Corporation). Root sections were prepared by sectioning perpendicular to the long axis at 2 mm intervals, creating 1 mm thick sections from coronal, middle and apical thirds of the post space. Push-out testing was performed at a crosshead speed of 0.5 mm/min using appropriately sized cylindrical plungers to avoid contact with dentin walls. Bond strength values were calculated using the formula: τ = F/(π x r x h), where F is the failure load in Newtons, r is the post radius and h is the section thickness. Results were expressed in megapascals (MPa) and analyzed separately for each root region.

## Fatigue testing:

Specimens allocated for fatigue testing were subjected to step-stress fatigue protocols using the chewing simulator [9, 13]. Initial loading began at 200 N for 10,000 cycles, with load increments of 100 N every 15,000 cycles until failure or completion of 1.5 million cycles. Failure was defined as visible crack formation, post debonding, or catastrophic fracture observable through continuous monitoring and trans-illumination [15, 18 and 19].

## Failure mode analysis:

Failed specimens were examined using stereomicroscopy (Leica M205 A, Leica Microsystems) at 40x magnification to classify failure modes. Failures were categorized as: Type I (adhesive failure between post and cement), Type II (cohesive failure within cement), Type III (adhesive failure between cement and dentin), Type IV (cohesive failure within dentin), or Type V (post fracture). Additional examination using scanning electron microscopy (SEM) was performed on representative specimens to characterize interfacial morphology.

## Statistical analysis:

Data were analyzed using SPSS software (version 28.0, IBM Corporation) with significance set at α = 0.05. Bond strength data were analyzed using three-way ANOVA with post type, aging protocol and root region as factors, followed by Tukey's post hoc comparisons. Fatigue data were analyzed using Kaplan-Meier survival analysis with log-rank tests for group comparisons. Chi-square tests were used to analyze failure mode distributions between groups.

## Results:

Bond strength results demonstrated significant effects of post type, aging protocol and root region (p < 0.001 for all factors). Table 1 (see PDF) presents the mean bond strength values for all experimental conditions.

Zirconia posts demonstrated significantly higher initial bond strength compared to fiber posts across all root regions (p < 0.001). However, both post types showed progressive bond strength degradation with aging protocols. Thermocycling alone resulted in 12-15% reduction in bond strength for fiber posts and 18-22% reduction for zirconia posts. The combination of thermocycling and mechanical loading produced the greatest degradation, with reductions of 18-21% for fiber posts and 33-37% for zirconia posts. Regional analysis revealed consistently higher bond strengths in the coronal third compared to middle and apical regions for both post types (p < 0.001). This gradient was maintained across all aging protocols, though the magnitude of difference decreased with increasing degradation. Fatigue testing results are summarized in Table 2 (see PDF), showing survival rates and mean cycles to failure for each group.

Kaplan-Meier survival analysis revealed significant differences between post types (p = 0.018) and aging protocols (p < 0.001). Fiber posts demonstrated superior fatigue resistance across all testing conditions, with 90% survival rates maintained through thermocycling and 80% survival with combined aging. Zirconia posts showed progressive degradation in fatigue performance with aging, reaching only 60% survival under combined thermocycling and mechanical loading conditions. The step-stress protocol revealed distinct failure patterns between groups. Fiber post failures typically occurred at loads between 800-1200 N, while zirconia post failures were observed at lower loads ranging from 600-1000 N after aging. Control specimens showed less variability in failure loads compared to aged specimens. Failure mode analysis revealed significant differences between post types and aging conditions (p < 0.001). Table 3 (see PDF) summarizes the distribution of failure modes across experimental groups.

Fiber posts demonstrated a predominance of cohesive failures within the cement layer under control conditions, shifting toward adhesive failures at the post-cement interface with aging. Zirconia posts showed more diverse failure patterns, with increased adhesive failures at the cement-dentin interface following aging protocols. Catastrophic dentin failures (Type IV) occurred more frequently with zirconia posts, particularly in control conditions, while post fractures were rare across all groups. SEM analysis revealed distinct interfacial morphologies between post types. Fiber posts showed evidence of micro-mechanical interlocking with the resin cement, while zirconia posts demonstrated primarily chemical adhesion. Aging protocols resulted in visible degradation of the hybrid layer formation and increased porosity at bonding interfaces for both post types [20].

## Discussion:

The results of this comprehensive *in vitro* study provide valuable insights into the comparative performance of zirconia and fiber posts under simulated clinical aging conditions. The findings challenge several assumptions regarding the superiority of high-strength ceramic posts and highlight the importance of considering long-term stability rather than solely initial mechanical properties when selecting post materials for clinical applications. The initial bond strength advantage observed with zirconia posts aligns with previous research demonstrating the superior mechanical properties of this material [2, 11]. The higher bond strength values can be attributed to the increased stiffness and surface energy of zirconia, which may facilitate better initial contact with the luting cement. However, the progressive degradation of this bond strength advantage under aging conditions reveals the vulnerability of the zirconia-cement interface to environmental stresses. The differential response to thermocycling between post types is particularly noteworthy and supports previous findings regarding the thermal incompatibility between high-modulus ceramics and resin cements [4, 8]. The coefficient of thermal expansion mismatch between zirconia and the resin cement likely contributes to the development of interfacial stresses during thermal cycling, leading to progressive bond degradation. In contrast, the more compatible thermal expansion characteristics of fiber posts with resin-based materials appear to provide better long-term stability [10, 12]. The superior fatigue resistance demonstrated by fiber posts contradicts the common assumption that higher strength materials necessarily provide better clinical performance. This finding is consistent with recent research emphasizing the importance of elastic modulus compatibility in post and core systems [14, 15]. The ability of fiber posts to flex under load and distribute stresses more favorably throughout the root structure appears to outweigh the mechanical property advantages of zirconia under cyclic loading conditions. The failure mode analysis provides crucial insights into the clinical implications of these findings. The predominance of restorable failures (Types I and II) with fiber posts, particularly under aging conditions, suggests better clinical prognosis in the event of restoration failure. Conversely, the increased incidence of adhesive failures at the cement-dentin interface and cohesive dentin failures with zirconia posts indicates a higher risk of catastrophic, non-restorable failures that may necessitate tooth extraction. The regional variation in bond strength observed in this study confirms previous reports of decreased bonding effectiveness in the apical regions of the post space. This gradient likely reflects differences in dentin characteristics, including tubule density and orientation, as well as challenges in achieving effective polymerization of resin cements in confined spaces with limited light access. The maintenance of this gradient across aging protocols suggests that these anatomical factors remain influential regardless of post material selection.

The step-stress fatigue protocol employed in this study provides a more clinically relevant assessment of post-performance compared to traditional static load testing [13, 14, 15, 16-17]. The observation that fiber posts maintain higher survival rates and more predictable failure loads under increasing stress conditions has important implications for clinical longevity. The greater variability in failure loads observed with aged zirconia posts suggests reduced reliability under varying clinical conditions. Several limitations of this study should be acknowledged. The use of extracted teeth, while providing standardized specimens, cannot fully replicate the complex oral environment, including the influence of periodontal ligament support and varying loading patterns. The relatively short-term aging protocols, while standardized according to established correlations, may not capture all long-term degradation mechanisms relevant to clinical practice. Additionally, the focus on maxillary canines may not be fully representative of the clinical scenarios where posts are most commonly employed. The clinical implications of these findings suggest that the selection of post materials should consider long-term stability and biocompatibility rather than solely initial mechanical properties. The superior fatigue resistance and more favorable failure modes of fiber posts, combined with their adequate bond strength under clinical conditions, support their continued use as first-line treatment for most clinical scenarios. However, the higher initial bond strength of zirconia posts may still be advantageous in specific high-stress situations where short-term performance is critical. Future research should focus on developing surface treatments and bonding protocols that can improve the long-term stability of zirconia posts while maintaining their mechanical advantages. Additionally, investigation of hybrid approaches, such as fiber-reinforced ceramic posts, may provide optimal combinations of initial strength and long-term stability [16]. Clinical studies with extended follow-up periods are essential to validate these *in vitro* findings and establish evidence-based guidelines for post selection in various clinical scenarios.

## Conclusion:

Fiber posts demonstrate superior long-term stability and fatigue resistance under dynamic aging conditions compared to zirconia posts. Zirconia posts, despite higher initial bond strength, show significant performance degradation over time. Clinicians should prioritize fiber posts for long-term restorations, with zirconia reserved for cases needing immediate high-strength support.
